# Negative Healthcare Impacts of Management of Presumed Early-Onset Sepsis in Moderate to Late Preterm Infants on Feeding, Jaundice, and Hospital Length of Stay

**DOI:** 10.3390/healthcare13020136

**Published:** 2025-01-13

**Authors:** Daniel Ng, David Tran, Rami Subhi, Wei Qi Fan

**Affiliations:** 1 Department of Medicine, The Royal Melbourne Hospital, 300 Grattan St., Parkville, Melbourne, VIC 3050, Australia; daniel.ng@mh.org.au; 2 Faculty of Medicine, Dentistry and Health Sciences, The University of Melbourne, Grattan St., Melbourne, VIC 3010, Australia; david.tran2@nh.org.au (D.T.); rami.subhi3@nh.org.au (R.S.); 3 Neonatal Unit, Department of Paediatrics, Northern Health, 185 Cooper Street, Melbourne, VIC 3076, Australia

**Keywords:** presumed early-onset sepsis, antibiotics, feeding, jaundice, healthcare

## Abstract

Background/Objectives: Early-onset sepsis in neonates is a potentially catastrophic condition that demands prompt management. However, laboratory diagnosis via cerebral spinal fluid and blood tests is often inconclusive, so diagnosis on the basis of clinical symptoms and risk factors is frequently required, and the majority of neonates treated with antibiotics for presumed early-onset sepsis (PEOS) do not have culture-proven sepsis. The management of such PEOS is mainly achieved via antibiotic therapy, which itself has adverse effects, creating a dilemma for clinicians in optimising healthcare. This study aimed to assess the impact of PEOS management on the common neonatal concerns of feeding tolerance, hyperbilirubinaemia, weight gain, and length of stay (LoS) in moderate to late preterm infants. Methods: A single-site, matched-cohort, retrospective study was performed on infants born between 32^+3^ and 36^+6^ weeks (2016 to 2019) admitted to the Neonatal Unit. PEOS infants on antibiotics (PEOS) were strictly matched by gestational age (±1 day) and birthweight (±5%) against a non-PEOS reference group (NPEOS). The key outcomes included the following: enteral feeding commencement and achievement; feeding intolerance (FI); phototherapy commencement and duration; antibiotic therapy duration; maximum bilirubin (MaxBili); LoS; and net postbirth weight gain. Results: There were no cases of culture-proven early-onset sepsis. PEOS (n = 185): NPEOS (n = 185) via multivariable analysis showed delayed enteral feed commencement (adjusted Odds Ratio [aOR]: 2.75; 95% confidence interval [CI]: 2.32, 3.27); there was no difference in FI, delayed onset of peak jaundice (aOR: 1.24; 95%CI: 1.12, 1.37), increased duration of phototherapy (aOR: 1.24; 95%CI: 1.10, 1.41), and increased LoS (aOR: 1.31; 95%CI; 1.02, 1.67). A univariate analysis also showed the following results (PEOS: NPEOS): no significant difference in MaxBili and delayed full enteral feed achievement (*p* = 0.010). Univariant or multivariable analysis showed no difference in irradiance levels. However, for NPEOS infants undergoing 0 or 1 phototherapy light treatment, there was an increased irradiance for PEOS (<0.001, 0.037, respectively). Conclusions: In moderate to late preterm infants, while PEOS diagnosis and management resolve the negative health impacts of potential sepsis, they are associated with negative healthcare outcomes on feeding, jaundice, and hospital length of stay.

## 1. Introduction

Neonatal sepsis is a major cause of death, especially in low-birthweight and preterm infants, with complications including septic shock, multi-organ failure, and death [1]. Early-onset sepsis typically occurs within 72 h of birth [2]. Diagnosis is difficult, with signs and symptoms not dissimilar from other causes of neonatal distress [3]. Laboratory testing of cerebral spinal fluid (CSF) or blood is frequently inconclusive. Consequently, the decision to prescribe antibiotics in this age group is based on the risk stratification of presumed early-onset sepsis (PEOS), which is often based on maternal and infant risk factors.

The rational use of antibiotics is balanced by the devastating consequences of missed and untreated neonatal sepsis, which continues to have a mortality rate of 3% [4]. Neonatal sepsis is mainly caused by the vertical transmission of pathogens from mother to infant. Infection can be caused by transmission from contaminated amniotic fluid or from maternal lower genital tract microbes during vaginal delivery [5]. Infection-causative bacteria include the following: Group B *streptococcus* (GBS), *Streptococcus pneumoniae*, *Streptococcus pyogenes*, *Escherichia coli*, *Klebsiella pneumoniae*, *Staphylococcus aureus*, *Pseudomonas aeruginosa*, and *Salmonella typhi* [6].

Consequently, the major maternal risk factors for the diagnosis of PEOS relate to the potential mode of bacteria transmission and include the following: a preterm rupture of membranes, amnionitis, meconium aspiration, fever, and untreated or partially treated GBS infection. Infant infection sites can include the skin (usually not consequential), blood (often undetected, potentially a negative culture bacteraemia), lungs, intestine, and renal system [2]. Infant risk factors are more general and include prematurity or low birthweight and low APGAR scores. Infant vital signs are important in the diagnosis of PEOS. There are multiple vital signs and symptoms that are considered, such as the following: fever and hypothermia, tachycardia or bradycardia, poor perfusion, respiratory symptoms, neurological symptoms, gastrointestinal signs, and skin findings [6].

The main component of PEOS management is antibiotic therapy, together with close attention to the additional common comorbidities of prematurity such as hypoglycaemia, jaundice, and feeding issues. In Australia, the most commonly used antibiotic combination for PEOS is benzylpenicillin and gentamicin [7], which differs from Europe, where the most common antibiotic combination is ampicillin and gentamicin [8]. In Australia, it has been reported that the duration of antibiotic therapy for neonatal sepsis is >24 h for 48% of infants and >48 h for 22% of infants [7].

While the prevalence of culture-proven early-onset sepsis (EOS) is low in well-resourced settings (0.49 cases per 1000 live births, [4]), antibiotics are prescribed for 3% of all newborns [4]. There is a wide variation between countries, but the threshold for antibiotic prescription tends to be lower in Australia. In all, 98% of neonates prescribed antibiotics do not have culture-proven EOS. This exposes a large number of infants with PEOS to early antibiotic therapy, increasing the risk of adverse healthcare outcomes [9], including antibiotic resistance, induced microbiome alterations [9], and impacts on the developing immune system [10]. In premature infants, prolonged antibiotic therapy has been associated with late-onset sepsis, necrotising enterocolitis, and even death [11]. A key objective in the management of preterm infants is the optimisation of feeding and growing. Feeding achievement is of particular concern, but, in preterm infants, early antibiotic therapy is associated with delayed feeding tolerance [12]. The mechanism for this is unclear [12], but feeding intolerance (FI) in preterm infants is associated with altered gut microbiome composition [13], and antibiotic administration in premature infants affects the differential composition of gut microbiota [14]. Understandably, such adverse outcomes have prompted antibiotic reduction strategies such as the EOS Risk Calculator screening tool [15].

In our neonatal unit (NNU), we care for moderate to late preterm and low-birthweight infants as well as manage common morbidities such as respiratory distress, hypoglycaemia, feeding intolerance, and jaundice. Very few studies have examined the healthcare burden of PEOS diagnosis and management in this group of preterm infants, who constitute by far the largest number of postbirth hospital admissions. We were interested in exploring whether the additional health burden of PEOS and its management had an impact on the outcomes of common morbidities and other key healthcare factors, such as weight gain and length of hospitalisation. We therefore carried out a retrospective study in which infants not confirmed of having an infection but classified as PEOS were compared to a strictly gestational-age-matched cohort of non-PEOS infants with similar comorbidities.

## 2. Methods

### 2.1. Study Design and Participants

A retrospective matched-cohort study was conducted on infants admitted to the NNU at The Northern Hospital (TNH) in Victoria, Australia, between January 2016 and December 2019. Cases were all moderate to late preterm infants (≥32 to <37 weeks’ gestation) who were commenced on empirical antibiotic therapy during NNU admission for PEOS. PEOS cases were strictly matched to a control infant not receiving any antibiotic therapy during NNU admission (NPEOS) to firstly within ±1 day of gestational age (GA) and then within 5% of birthweight. A key aspect of selecting the PEOS infants admitted to the NNU was to provide comparability with the burden of morbidity and patient complexity. Given the constraints of the PEOS-classified infants and controls available from the TNH Birth Register during this period of time, the final analyses included 185 matched PEOS and NPEOS pairs and infants between 32^+3^ and 36^+6^ weeks gestation—all meeting the inclusion criteria.

TNH serves a lower socioeconomic, multicultural, and multiethnic community.

Inclusion criteria were as follows: infants requiring NNU admission at birth and born at less than 37 weeks’ gestation. Infant exclusion criteria were as follows: neonatal death (within the first 28 days of life); recognised chromosomal abnormalities; transferral to tertiary hospitals shortly after birth.

### 2.2. Ethical Approval

This study was performed in line with the principles of the Declaration of Helsinki. Ethics approval was provided by the Northern Health Office of Research (ALR No. 51.2019). As this was a retrospective study with de-identified patient data, the Northern Health Office of Research considered informed consent from participants as not applicable.

### 2.3. Data Collection and Outcomes

The study cohort was selected from a database of births at TNH provided by the Northern Health Decision Support Department.

The primary outcomes were neonatal hyperbilirubinaemia and feeding tolerance—as defined below.

Additional hyperbilirubinaemia outcomes were as follows: maximum total bilirubin concentration (µmol/L); age in hours at maximum total bilirubin concentration; maximum direct bilirubin concentration (µmol/L); maximum number of phototherapy lights (single, double, or higher); total hours of phototherapy. Phototherapy was provided via a MediLED Mini (Medix, Jose Arias 293, Villa Lynch San Martin, 1672 Buenos Aires, Argentina). Quality control was managed routinely by the hospital’s Biomedical Engineering Department. Phototherapy requirement was determined via “Treatment for Hyperbilirubinaemia in Newborn Babies Charts” specific for gestational age and bilirubin level, developed as a guideline policy by the Royal Women’s Hospital, Melbourne, Australia.

Additional enteral feeding outcomes were as follows: admission enteral feeding type (exclusively breastmilk, formula, mixed feeds); hours to commence enteral feeding; days to reach full enteral feeding (total fluid intake of ≥150 mL/kg/day).

Secondary outcomes were the following: NNU length of stay in days (LoS); growth as defined by the postbirth net gain in weight (discharge minus birthweight); weight Z score gain on discharge (adjusted for gestational age and sex using 2013 Fenton growth charts [16]).

### 2.4. Definitions

Definitions for the purposes of this study were as follows: PEOS was presumed sepsis as not confirmed by blood or CSF culture but via clinical suspicion within 72 h of birth; clinical suspicion was determined by the clinician and encompassed maternal and infant infection risk factors, clinical assessment of unwellness and response to laboratory blood and CSF findings (the antibiotic duration for PEOS was determined depending on clinical assessment and laboratory findings. Antibiotic therapy typically varied between 36 h and 7 days); EOS was early-onset sepsis infants confirmed to have a bacterial infection by blood or CSF culture; respiratory distress occurs in premature newborns due to breathing difficulties often as a result of lack of surfactant. Diagnosis was made on the basis of clinical findings such as nasal flaring and grunting and/or radiological findings. (Management strategies included the following: respiratory support; oxygenation; temperature maintenance; antibiotics; surfactant administration; neonatal hyperbilirubinaemia requiring phototherapy for jaundice management); feeding tolerance was determined as the preterm infant being able to safely ingest/digest prescribed enteral feeding without complications associated with aspiration, infection, and gastrointestinal dysfunction; FI was significant abdominal distention and/or emesis in the neonate resulting in a lack of feeding tolerance.

### 2.5. Sample Size Calculations

We knew from in-house audits that the frequency of FI in the NPEOS group was around 10%. We wanted to test for an increase in FI in the order of between 10% and 15%. In each group, 12.5% gave us a sample size of 136, 12.9% a sample size of 128 and 13.4% a sample size of 120, where the power was 80%, and alpha 0.05. We chose 128 in each group but aimed to keep numbers in this study above this estimate. Please see the definitions for the criteria for FI assessment and the subsequent record keeping.

### 2.6. Statistical Analysis

Data were analysed using IBM SPSS Statistics for Windows, Version 26 (IBM Corp., Armonk, NY, USA) and Stata Statistical Software for Windows, Release 16 (StataCorp LLC., College Station, TX, USA). Assistance was provided by the TNH research statistician. Parametric and nonparametric tests were used to determine the normality of continuous variables. For univariate analysis, chi-square and Fisher’s exact tests were used for categorical factors. Mann–Whitney rank-sum and Kruskal–Wallis H tests were used for continuous factors.

Confounding variables were identified as factors with *p* < 0.2 through univariate analysis plus factors considered likely to be clinically relevant. For all outcomes (except for the time to reach full enteral feeding), a process of backward and forward multivariable logistic regression was used (stepwise regression) [17]. For the time to reach full enteral feeding outcomes, Cox regression time-to-event analysis was used.

Significance was determined via two-tailed *p*-values < 0.05 for univariate and multivariable analyses.

## 3. Results

### 3.1. Overview

**Figure 1:**
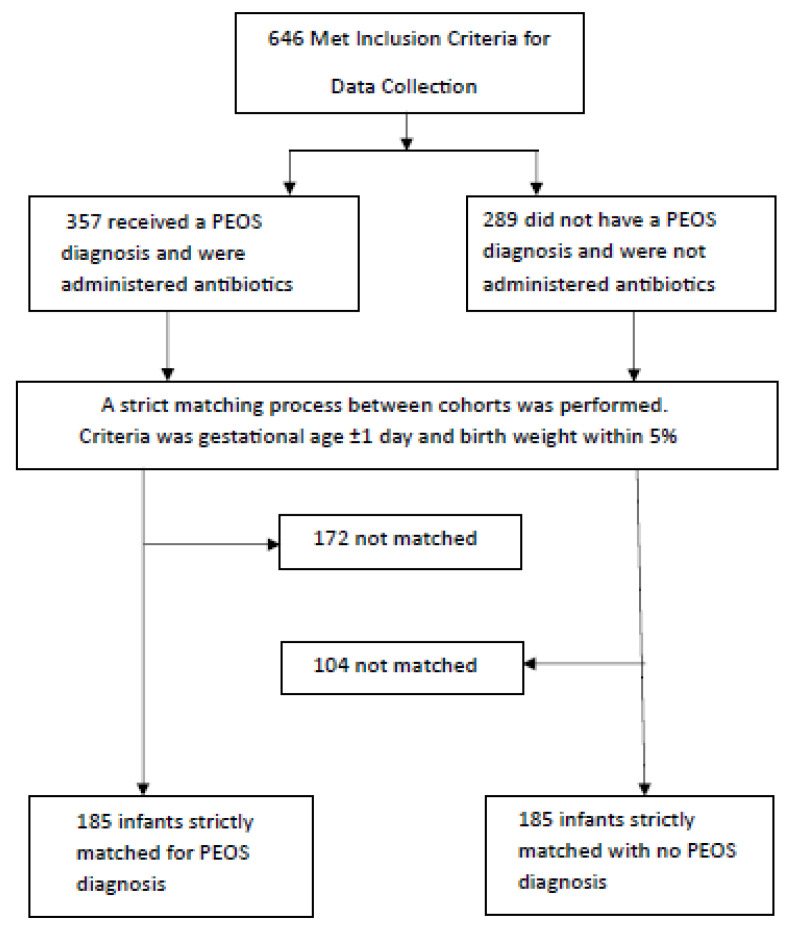
Flow chart of study cohort selection.

A total of 370 preterm infant–mother pairs were enrolled and separated into the PEOS group (n = 185) or NPEOS group (no antibiotic therapy) (n = 185) during NNU admission. Figure 1 illustrates the cohort selection process. The univariate analysis results are presented in Table 1, Table 2 and Table 3 and multivariable analysis in Table 4.

### 3.2. Antibiotic Therapy

All 185 infants included in this study were given antibiotics to control potential sepsis. The great majority (n = 169) were given a combination of benzylpenicillin (Gram-positive) and gentamicin (Gram-negative) only. Eleven infants were given an additional narrow-spectrum antibiotic (flucloxacillin or metronidazole). Broad-spectrum antibiotics were used in approximately 3% of infants—cefotaxime (four infants) or ampicillin (one infant) combined with the narrow-spectrum antibiotics benzylpenicillin and gentamicin.

### 3.3. Baseline Characteristics—Table 1

The key factors of maternal age, neonatal birthweight, as well as gestational age and sex were all matched statistically. In the PEOS group, there was a higher frequency of maternal intrapartum fever, neonatal vaginal birth, instrument delivery, birth trauma, resuscitation, and respiratory distress (a number of these factors are prompts for the suspicion of PEOS). All of the PEOS group had blood- or CSF-culture-negative status.

### 3.4. Jaundice Outcomes—Table 2

There was no evidence of a difference in the maximum total direct bilirubin. The peak jaundice level occurred approximately 20 h later in the PEOS group.

The duration of phototherapy was almost 10 h longer for the PEOS group.

When the NPEOS group was used as a baseline for those infants having no jaundice or jaundice requiring only one phototherapy light session, there was a bias towards increased irradiance (light number exposure) in the PEOS group.

### 3.5. Feeding Tolerance and Secondary Results—Table 3

There was no difference in the FI between the groups.

For the PEOS group, median enteral feed commencement was delayed by 1 h (IQR 8.5 vs. 0.6 h for NPEOS). Median full enteral feeding achievement was delayed by 2 days.

The median LoS was approximately two days longer for the PEOS group compared to the NPEOS group.

### 3.6. Multivariable Analysis—Table 4

Many of the findings in the univariate analysis were also found to be significant in the multivariable analysis. These included, for the PEOS group compared to NPEOS group, a delayed onset of jaundice, a greater duration of phototherapy, a delayed time to commence enteral feeding, a longer time achieve full enteral feeding, and a longer LoS.

## 4. Discussion

The key healthcare findings of our study via univariate and multivariable logistic regression were that management for PEOS in moderate to late preterm infants was associated with a delay in enteral feeding commencement, a delay in reaching full enteral feeding, increased LoS, a delayed onset of peak jaundice by around 20 h, and an increased duration of phototherapy. The findings regarding jaundice appear to be previously unreported.

Importantly, none of the infants in either the PEOS or NPEOS group had culture-proven EOS. While we could not estimate the prevalence of culture-negative EOS from a retrospective study reliant on medical documentation, it is clear that our study aligns with the previous literature showing the overdiagnosis of PEOS and overtreatment with antibiotics [4]. These findings have multiple healthcare implications. There are negative impacts both on the infants (increased burden of illness, potential breastfeeding disruption), the mothers (disruption of mother–child bonding and family dynamics), the hospital healthcare system (higher costs and more resources required for nursing, equipment, maintenance), and on the community (impact on community child health resources, local general and specialist practices, private and government funding). These impacts are further discussed below in relation to hyperbilirubinaemia management.

Our study cannot prove causation between antibiotic exposure and adverse patient outcomes. However, it is notable that our management of PEOS, apart from antibiotic therapy to overcome an anticipated infection and close attention to the management of co-morbidities, differs little from that for any other moderate to late term infant—the key objectives being feeding and growing. There is evidence that antibiotic therapy impacts feeding and LoS in very preterm infants, term infants, and animal studies. Given the importance of nutrition, there have been a number of studies on antibiotic use and feeding implications in very-low-birthweight infants (birthweight < 1500 g). Ampicillin and gentamicin delayed feeding initiation and total milk intake and increased LoS [11]. Antibiotic therapy prolonged total parental nutrition [12]. Early antibiotics delayed the achievement of milk feeding, but antibiotics commenced 9 days after birth reduced this delay [18], suggesting that early antibiotics interfere with gastrointestinal (GI) tract maturation. In the term infant, early ampicillin and gentamicin resulted in increased and persistent levels of potentially pathogenic Proteobacteria and decreased beneficial *Actinobacteria, Bifidobacteria,* and *Lactobacillus* [19]. Animal studies have shown that the postnatal maturation of the intestine is partially modulated by bacterial colonisation establishing a barrier to luminal antigens [20]. Gut immunological, sensory, and motor functions in the immature GI tract are developed and maintained by commensal bacteria, with phyla including *Bifidobacterium* and *Lactobacillus* enhancing intestinal epithelia survival [21]. Consequently, antibiotic-altered GI microbiota potentially disrupt the development of GI immune and neural patterns, contributing to the aetiology of feeding dysfunction [21].

There is also evidence to support the contention that antibiotic therapy in PEOS infants influenced this study’s observation of a later shift in peak bilirubin and increased irradiation. This evidence relates to the chemical impact of antibiotics on the red cell membrane and their physiological impact on the gut. Following birth, red blood cells break down, then haem degradation occurs via haem oxygenase to biliverdin and then bilirubin [22]. The steep rise in bilirubin is due to the shortened life span of erythrocytes in full-term and preterm infants. Eighty to ninety percent of bilirubin is formed from haemolysed or senescent erythrocytes [22]. Antibiotics strengthen the erythrocyte membrane, leading to a reduction in haemolysis. Penicillin and gentamicin have both been shown to decrease haemolysis in a dose-dependent manner [23]. In the case of benzylpenicillin, increased cellular potassium levels are implicated in this strengthening of the erythrocyte membrane [24]. Consequently, it is plausible that the observed PEOS vs. NPEOS peak bilirubin delay is a result of delayed levels of initial erythrocyte haemolysis due to antibiotic red cell membrane strengthening. Another aspect that may impact bilirubin conjugation and elimination relates to the potential for antibiotics to negatively impact the immature neonatal gut. Bilirubin elimination is a complex process requiring conjugation and gut elimination. UDP-glucuronosyltransferase 1A1 (UGT1A1) is solely responsible for bilirubin conjugation and is expressed in both the liver and intestine [25]. In the gut, UGT1A1 is induced by enteral feeding and especially glucose (e.g., starch maltodextrin as present in milk fortifiers) and has the potential to be very effective as it can re-conjugate bilirubin that has been de-conjugated by the β-glucuronidase present in breast milk [26] and in gut bacteria [27], enhancing bilirubin elimination and enterohepatic re-circulation [25]. We hypothesise that the increase in PEOS duration of phototherapy is potentially related to antibiotic gut dysfunction that delays enteral feeding [11,18], thus downregulating intestinal UGT1A1 expression and burdening liver UGT1A1.

Our finding that the moderate to late preterm infant may have delayed and more intractable jaundice is important as hyperbilirubinaemia is such a common neonatal healthcare concern. Apart from the individual healthcare concerns for the infant due to phototherapy, the hospital/healthcare system is impacted as more resources are required for medical equipment and medical consumables, and funding is required for a potential delay in discharge. While the immediate phototherapy management ramifications include insensible water loss and increased peripheral blood flow, there are potentially medium-term healthcare implications both for the patient and the healthcare system due to the association of phototherapy with childhood type 1 diabetes and asthma [28].

It is possible that our findings reflect PEOS infants being potentially unwell compared to the comparison cohort rather than a more specific influence due to antibiotic therapy itself. There is mixed evidence in Table 1 regarding whether PEOS infants may have been more compromised than the gestationally age-matched cohort. This evidence relates to maternal intrapartum fever, birth mode, neonatal birth trauma, resuscitation, and respiratory distress. Maternal intrapartum fever is a risk factor for the suspicion of neonatal infection and is strongly associated with neonatal antibiotic therapy [29]. However, much of the fever is due to epidural anaesthesia and not maternal infection, with the effects of fever, such as neonatal resuscitation (also evident in this study), being transient [30]. The higher rate of normal vaginal births in the PEOS group was clearly not an indicator of comparatively unwell infants but again is a risk factor for PEOS management due to the possibility of vertical infection transmission during the birth process [5]. Countering this aspect was the higher rate of emergency Caesarean births in the NPEOS group due not only to maternal concerns but also the infant’s well-being. Neonatal birth trauma was higher in the PEOS group. Bruising from such trauma would be expected to result in an earlier presentation of jaundice due to the breakdown of red blood cells, but we observed the opposite effect in our study. Respiratory distress was higher in the PEOS group, suggesting a bias towards sicker infants—this is an expected result, as respiratory distress is a strong risk factor for PEOS management due to the possibility of infection. However, Table 1 findings do not provide general evidence to support more compromised infants in the PEOS group—there was no difference between the cohorts regarding maternal white cell count (WCC) or C-reactive protein (CRP) or neonatal hypoglycaemia, haemolysis, birth temperature, WCC, or CRP. Additionally, there was a high probability that many in our cohort of PEOS infants did not have an infection and were not relatively unwell. For preterm neonates in Australia, it is mandatory to have a high index of suspicion for the possibility of sepsis as well as a low threshold for commencing antibiotic treatment, with the consequence being that more babies are treated than actually have an infection [31]. This local protocol is corroborated by a recent study comparing centres across many countries. The Australian use of antibiotic therapy for infants within the first postnatal week of life with EOS or PEOS was 12.5%. This was almost three times higher than that of the nearest other country [4]. Supporting the contention that many of our infants did not have sepsis is the short duration of antibiotic treatment evidenced in Table 1. The median duration of antibiotic treatment was 36 h (i.e., 50% of the cohort), which is the minimal amount of antibiotic exposure in our treatment regime, suggesting that clinicians were confident in ruling out infection as antibiotic therapy was not extended. Many of these infants, rather than being classified as “culture-negative sepsis”, could arguably be referred to as “sepsis ruled out”. Consequently, it was difficult to determine the unwellness status of either the PEOS or NPEOS groups. However, it should also be noted that the multivariable regression analysis presented in Table 4 was adjusted for potential confounders that included variables contributing towards unwellness. It should be noted that it was not possible in this retrospective study to directly attribute an outcome to antibiotic therapy. This was in part due to the inability to control intangibles in the management of PEOS relating to a clinician’s suspicion of infection and subsequent responses to management such as timing of antibiotic cessation and the caution of longer monitoring.

Reducing antibiotic use via a stewardship scheme is an approach that has the potential to moderate negative clinical and healthcare impacts. The Kaiser Permanente early-onset sepsis calculator (EOSC) was developed in the U.S. for U.S. conditions and allows an objective approach to the decision on whether to prescribe antibiotics [15]. The calculator has a number of fields such as community EOS incidence, gestational age, and maternal aspects (highest antepartum temperature, timing of rupture of membranes, GBS status, timing of intrapartum antibiotics) and derives a risk factor that guides antibiotic use or continued observation. Its introduction has been documented to reduce antibiotic therapy for EOS. However, there are concerns that this calculator has limitations when used outside of the U.S. For example, in the U.K., its introduction has not been recommended because of differences between the two countries in microbiology and healthcare practices and a resulting large proportion of missed EOS cases especially in mothers with chorioamnionitis [32]. As discussed above, the mandatory requirements of local PEOS antibiotic therapy in Australia result in a higher proportion of infants receiving antibiotics than other high-income countries [4], with the implication being that many of these infants do not have an infection, are being treated unnecessarily, and that there is considerable scope for a marked reduction in EOS antibiotics. However, in Australia, there has been limited experience and mixed results with the EOSC, indicating that the EOSC algorithm needs local recalibration. One site reported a significant drop in antibiotic use overall [33], while another site reported an increase in missed EOS cases [34], and another site found no difference in PEOS antibiotic use [35]. In the study reporting missed cases of EOS [34], it was speculated that the missed cases were influenced by differences in GBS testing rates and antepartum antibiotic administration rates as well as “antepartum temperature” rather than a more appropriate “maternal fever” being a field in the calculator. To this end, our hospital introduced the EOSC in late 2020. Given the level of uncertainty in its use in Australia, it is being used as a screening tool only. We anticipate that on the completion of 5 years of EOSC use, we will carry out a detailed review study with a comparison to the pre-EOSC data from this current study.

Our study has limitations in that it is retrospective in nature. It had to rely on data routinely collected and not subjected to the rigours of a controlled trial. Additionally, there were multiple potential confounders that impact the verification of the findings. As mentioned above, there were intangibles that were difficult to control in the management of PEOS such as reluctance to push feeding in an infant perceived as ‘sick’, clinical discretion, and level of experience. Clinician ‘fear’, the time preference of being concerned about an infant’s condition in the present, rather than consideration of potential later negative sequalae and just simply ‘noise’, have been identified as challenges in the decision-making process [9]. Despite these intangibles, the strengths of this study relate to how these potential negative aspects were accounted for. As a matched-cohort study, matching was performed on the basis of confounders such as gestational age, birthweight, and sex—an approach that improved prediction but could not necessarily prove cause-and-effect relationships [36]. Matching gestational age as a confounder is particularly important because, in preterm infants, it is the strongest single predictor of infection [37]. Regarding the key findings relating to jaundice and feeding, in addition to gestational age, the matching of birthweight is also important, as jaundice frequency and levels are negatively correlated to both gestational age and weight in preterm infants [38,39], and birthweight is positively associated with feeding strength and intensity [40]. Additionally, in this study, we rigorously applied stepwise multivariable logistic regression (variables retained or removed are listed as a footnote to Table 4). In a retrospective study, when there are potential confounders, multivariable analysis provides the only solution for control [41].

## 5. Conclusions

This study raises concerns that the diagnosis and management for PEOS are associated with negative healthcare outcomes such as feeding dysfunction and jaundice exacerbation in the moderate to late preterm infant and importantly increase hospital length of stay—the latter increasing the potential for further infant infection and increasing healthcare costs. Furthermore, we highlight the concerns of antibiotic overtreatment of PEOS and the need for improving systems for rationalising decision making for antibiotic prescription including alternative infection assessment algorithms such as the EOSC, which need to be fine-tuned for local conditions.

## Figures and Tables

**Table 1:** Maternal and infant cohort comparisons—NPEOS vs. PEOS.

	NPEOS (n = 185)	PEOS (n = 185)	*p*-Value
Maternal characteristics			
Age median (IQR) (years) (paired)	30 (7)	30 (7)	0.288
Region of birth, %			
Australia, New Zealand vs. other (paired)	51.9%	54.1%	0.755
Gravida, median (IQR) (paired)	2 (2)	2 (2)	0.658
Para, median (IQR)	1 (1)	1 (1)	0.790
GBS-positive status	22.0%	25.2%	0.645
Maternal intrapartum fever	0%	5.9%	0.001
Maternal intrapartum antibiotics	14.1%	20.5%	0.130
WCC between 6 and 15 × 10^9^/L	82.4%	84.8%	0.571
Maternal CRP < 5, mg/L	38.5%	25.1%	0.112
Neonatal characteristics			
Gestational age, median (IQR) (weeks) (paired)	35.5 (1.3)	35.4 (1.3)	0.194
Birthweight, mean (SD) (grams) (paired)	2364 (454)	2365 (451)	0.958
Male, %	53.5%	58.4%	0.402
Apgar 5 min, median (IQR) (unpaired)	9 (0)	9 (0)	0.030
Foetal distress, %	35.7%	35.7%	1.000
Delivery mode, %			
Normal vaginal delivery	34.1%	63.8%	<0.001
Emergency Caesarean	43.8%	30.8%	0.013
Elective Caesarean	22.2%	5.4%	<0.001
Instrumentation at delivery, %	11.4%	14.1%	0.533
Neonatal birth trauma, %	2.7%	14.1%	<0.001
Neonatal resuscitation, %	28.6%	42.2%	0.009
Neonatal respiratory distress, %	29.7%	53.5%	<0.001
Neonatal hypoglycaemia, %	35.7%	31.4%	0.441
Haemolysis present, %	3.2%	2.3%	0.716
Birth temperature, mean (SD) °C (unpaired)	36.5 (0.37)	36.6 (0.47)	0.062
WCC between 5 and 30 × 10^9^/L	2.2%	2.0%	1.000
CRP < 10 mg/L	96.6%	91.7%	0.166
Neonatal blood culture positive	NIL	NIL	-------
Antibiotic duration, median (IQR), hours	------	36 (12)	-------

Chi-square test for categorical variables, Student’s *t*-test or Mann–Whitney U test. Maternal CRP—normal reference values < 5 mg/L. Neonatal CRP normal reference range < 10 mg/L Maternal WCC—white cell count normal reference range 6–15 × 10^9^/L. Neonatal WCC—normal reference range 5–30 × 10^9^/L.

**Table 2:** Neonatal jaundice.

	NPEOS	PEOS	*p*-Value
Neonatal jaundice outcomes			
Any phototherapy	75.2%	76.5%	0.806
Maximum total bilirubin, mean (SD) (µmol/L)	213 (45)	214 (46)	0.840
Maximum direct bilirubin, mean (SD) (µmol/L)	12 (4)	11(4)	0.398
Age at maximum total bilirubin, median (IQR) (hours of life)	72 (51)	93 (55)	0.002
Phototherapy treatment duration, median (IQR) (hours)	28 (22)	38(26)	0.016
Phototherapy light dosage	Number of light dosages		
Median (IQR) [paired, n = 20]	0 (0)	1.5 (1.25)	<0.001
Median (IQR) [paired, n = 18]	1 (0)	2 (1.75)	0.037
Median (IQR) [paired, n = 27]	2 (0)	2 (3)	0.085
(NPEOS infants compared to PEOS gestation-matched infants)			

Maximum total bilirubin (paired, n = 86); maximum direct bilirubin (paired n = 87); age at maximum total bilirubin (unpaired, NPEOS n = 125, PEOS n = 132); phototherapy treatment duration (unpaired, NPEOS n = 93, PEOS n = 100).

**Table 3:** Feeding and secondary outcomes.

	NPEOS	PEOS	*p*-Value
Feeding intolerance %	10.8%	10.8%	1.000
Enteral feed during admission			
Breast milk: exclusive %	4.3%	8.6%	0.138
Mixed %	87.0%	82.2%	0.249
Enteral feed commencement, median (IQR) (hours)	1.0 (0.6)	2.0 (8.5)	<0.001
Full enteral feed achievement, median (IQR) (days)	6 (1.0)	8(12)	0.010
Secondary outcomes			
LoS, median (IQR) (days)	8 (12)	10 (11)	0.002
Readmission %	13.0%	13.0%	1.000
Weight gain from birth at discharge in g, mean (SD)	81 (279)	116 (271)	0.031

Enteral feeding during admission (n = 185); LoS (paired n = 184); enteral feeding commencement (unpaired n = 185); feeding intolerance (n = 185); full enteral feeding (unpaired n = 130 (NPEOS), n = 185 (PEOS); weight gain (paired n = 185); LoS—length of stay in NNU.

**Table 4:** Multivariable regression analysis for PEOS infants compared to the NPEOS group (n = 370).

Outcome	Odds or Hazards Ratio	95% Confidence Interval	*p*-Value
Neonatal hyperbilirubinaemia/phototherapy *	1.04	0.77–1.4	0.791
Triple phototherapy *	1.54	0.65–3.65	0.325
Double phototherapy *	0.92	0.39–2.19	0.853
Duration of phototherapy *	1.24	1.10–1.41	0.001
Age at maximum bilirubin *	1.24	1.12–1.37	0.001
Feeding intolerance †	1.00	0.52–1.93	1.000
Time to commence enteral feeds †	2.75	2.32–3.27	<0.001
Time to reach full enteral feeds †	1.10	0.96–1.25	0.173
Length of stay ‡	1.31	1.02–1.67	0.035
Readmission ‡	1.65	0.65–4.16	0.290
Weight gain on discharge ^	1.10	0.87–1.38	0.433
Weight Z-score gain on discharge ^	0.96	0.89–1.04	0.303

In all cases, statistical significance (<0.05) indicated a compromised outcome for the PEOS group. For the time to reach full enteral feeding, a time-to-event analysis using Cox regression was used. These results are presented as hazard ratios with 95% confidence intervals. All other results are presented as odds ratios with 95% confidence intervals. * Variables included use of maternal intrapartum antibiotics, mode of delivery, neonatal hypoglycaemia, birth trauma, maternal blood group, neonatal blood group, gestational age, birthweight, maternal WCC count, neonatal temperature. † Variables included gestational age, birthweight, maternal intrapartum fever, use of maternal intrapartum antibiotics, use of maternal analgesia, foetal distress, birth trauma, neonatal resuscitation, respiratory distress, neonatal hypoglycaemia, neonatal temperature. ‡ Variables included maternal ethnicity, maternal blood group, maternal analgesia, neonatal hypoglycaemia, neonatal blood group, foetal distress, mode of delivery, use of instrumentation during birth, birth trauma, neonatal resuscitation, respiratory distress, gestational age, maternal age, birthweight, APGAR 1 and 5 min. ^ Variables included maternal fever, neonatal hypoglycaemia, birth trauma, neonatal temperature, foetal distress, use of maternal intrapartum antibiotics, mode of delivery, respiratory distress, gestational age.

## Data Availability

The data that support the findings of this study are not publicly available due to their containing information that could compromise the privacy of research participants but are available from the corresponding author upon reasonable request.
